# Patients’ opinions about referral from a tertiary specialist psychiatric hospital to primary healthcare

**DOI:** 10.4102/sajpsychiatry.v25i0.1212

**Published:** 2019-01-31

**Authors:** Rene Hattingh, Pierre M. Joubert

**Affiliations:** 1 Department of Psychiatry, School of Medicine, Faculty of Health Sciences, University of Pretoria, South Africa

## Abstract

**Background:**

Referral of patients from tertiary specialist psychiatric hospitals to primary healthcare settings is a worldwide goal. This is of particular importance in South Africa with its considerable burden of mental disorders and limited resources. However, patients are often reluctant to be referred and studies have shown that patients may prefer a dedicated psychiatric service over an integrated primary healthcare service.

**Aim:**

This study explored the opinions of patients receiving care at a tertiary psychiatric hospital’s outpatient department (OPD) about referral to a primary healthcare clinic (PHCC).

**Setting:**

The study was conducted at Weskoppies Psychiatric Hospital OPD.

**Methods:**

This was a qualitative study based on grounded theory. Participants were recruited through purposive-theoretical sampling. Data were collected by means of individual interviews and mini-essays.

**Results:**

From the 80 participants, 18 had individual interviews and 62 wrote mini-essays. Thirty-nine participants had previously attended a PHCC, while 41 had not. Perceived advantages of referral to PHCCs included: close proximity to participants’ homes, resulting in saving on travelling time and transport costs, as well as the convenience of receiving psychiatric and other medical treatment at the same healthcare facility. Perceived disadvantages of PHCCs included: unavailability of treatment; lack of doctor-based care; lack of specialised care; loss of established relationships with hospital healthcare workers; mistreatment by PHCC nursing staff; longer waiting times; more stigmatisation.

**Conclusion:**

The perceived disadvantages of referral from a tertiary psychiatric hospital to a PHCC outweighed the perceived advantages. Nonetheless, participants expressed willingness for such a referral if their concerns were addressed.

## Introduction

It is a worldwide goal to shift from centralised mental healthcare services (provided by specialist psychiatric hospitals) to integrated, community-based services.^1,2^ This goal is very relevant to mental healthcare in South Africa, with its high prevalence of mental disorders and limited resources.^3,4,5^ In the most recent World Health Organization (WHO) Mental Health Atlas country profile, the burden of mental disorders in South Africa is estimated at 3.191 disability-adjusted life years per 100 000 population. South Africans suffering from mental disorders are served by an estimated 0.4 psychiatrists per 100 000 of the population.^6^ There are only 63 specialist tertiary healthcare institutions.^6^ Although specialist services are needed, research indicates that the most cost-effective interventions involve incorporating mental healthcare into primary healthcare.^1,2,3,7^

Referral of patients from tertiary specialist psychiatric hospitals to primary healthcare settings is advocated not only by WHO guidelines^1,2^ but also by South African legislation and policies.^3,8,9,10,11^ Doing so is based on the following presumed advantages: patients can access mental health services closer to their homes (thus avoiding costs associated with seeking specialist care in distant locations); moreover, mental health services delivered in primary care may minimise stigmatisation and discrimination and ensure holistic treatment that addresses both physical and mental healthcare needs.^1,2^

This model of care acknowledges that patients may at times require secondary- and tertiary-level psychiatric interventions but advocates the subsequent referral to primary level care for ongoing management.^2,5^

However, decentralisation of mental healthcare requires the public health sector to ensure that primary healthcare clinics (PHCCs) have adequate infrastructure, staff, expertise and resources to provide the necessary care for patients.^1,2^ The National Mental Health Policy, the South African Society of Psychiatrists, various patient advocacy groups, researchers and other stakeholders have expressed concerns about the lack of development of adequate primary healthcare services in concurrence with downscaling of hospital-based care.^3,12,13,14,15^

Furthermore, there are studies that show that in South Africa mental health patients are often reluctant to be referred to a primary health facility. Such studies show that patients prefer a dedicated psychiatric service over an integrated service at primary healthcare level.^4,16,17^

Both the WHO and the South African government promote the active involvement of patients in the reorganisation, delivery, evaluation and monitoring of mental health services, so that care and treatment can become more responsive to patient needs.^1,2,3^

Consequently, going forward, research on patients’ opinions and experiences are essential to inform decision-makers on policies and the provision of mental healthcare within the general healthcare system. Unfortunately, despite the need for it, such research is scarce.

## Research methods and design

### Study design

A qualitative study design based on grounded theory was followed.^18,19^

### Setting

Weskoppies Hospital (WKH) is a tertiary specialist psychiatric hospital situated in Pretoria West, Gauteng, South Africa. It forms part of the public healthcare system and is affiliated to the University of Pretoria, Sefako Makgatho Health Sciences University and Nursing Colleges. With its general adult psychiatry units, a substance rehabilitation unit, child and adolescent unit, forensic psychiatry unit and daily outpatient clinics, WKH serves Tshwane, Metsweding, Thembisa and Mpumalanga Province.

### Study population and sampling

Participants were selected by following a qualitative process-evaluation design with purposive-theoretical sampling.^18^ Consequently, every consentable outpatient who attended WKH outpatient department (OPD) between October and December 2016, who was willing to participate and who met the inclusion criteria of the study, was included. For inclusion in the study, participants had to be (1) 18 years or older, (2) able to communicate in Afrikaans or English, and (3) able and willing to give informed consent.

### Data collection

Participants could choose to participate in individual interviews or to write a mini-essay. A semi-structured guide was followed for conducting individual interviews (guide available from authors on request). Participants were allowed to elaborate freely on every question. Interviews were recorded in MP3 format and later transcribed into written format. Participants who chose the mini-essays were guided by open-ended questions similar to the questions of the individual interviews to which they could respond in their own way. Data were captured from the transcribed MP3 recordings of interviews, the researcher’s field notes and the mini-essays written by participants.

### Data analysis

Data analysis was guided by grounded theory, using a thematic content analysis. A coding scheme was used to identify the themes evident from the data.^13,18^ The first author extracted the information from the data sources. Similar themes were allocated to a specific category that captured the same idea. These categories were compared and further allocated to overall categories. To increase validity, the second author scrutinised the extracted information and themes. The authors then discussed the findings. After reaching consensus regarding themes and categories, they were then categorised into two overall categories that emerged from the data, namely advantages and disadvantages of referral to a PHCC.

## Ethical consideration

Ethical approval to conduct this study was obtained from the Research Ethics Committee of the Faculty of Health Sciences, University of Pretoria (No. 59/2015). Participation in the study was voluntary and participants were informed that they could withdraw from the study at any point. An information leaflet about the study’s objectives was provided to all the participants in Afrikaans and English.

## Results

Eighty participants were recruited into the study. Thirty-nine of them had experience of referral to a PHCC, whereas the remaining 41 had not. Eighteen participants chose to do individual interviews, while 62 chose to write mini-essays.

Data analysis revealed two main overall categories that had been grounded in participant responses: *perceived advantages* and *perceived disadvantages* of referral to a PHCC. There was also a third, smaller, but nonetheless significant category, namely *willingness to be referred to a PHCC if concerns are resolved*.

## Perceived advantages of referral

Two categories contributed to the overall category of perceived advantages of referral to a PHCC, namely that PHCCs are closer to patients’ homes and that it is convenient to receive psychiatric and medical treatment at one service delivery point.

### Saving on time and money

Primary healthcare clinics (PHCC) are in the community and thus closer to participants’ homes. It therefore comes as no surprise that participants generally thought that referral to a PHCC would save time and money:

‘I think it is good to be referred to a clinic for treatment because it will help you to save the money that you use when coming to hospital. It will benefit me because I can walk to the nearest clinic to collect my treatment.’ (Participant 29, mini-essay)

### The convenience of receiving psychiatric and medical treatment at one service delivery point

Participants with comorbid medical illnesses expressed the desire to get all their treatment at one service delivery point. Because only psychiatric treatment is dispensed at WKH OPD, patients need to attend a PHCC for their medical treatment. Thus, attending a PHCC could be advantageous, because:

‘I can only get my psychiatric treatment here [*WKH*]. So, I need to go to my clinic for my diabetes medication. It would be much better if I could get all my treatment at the same place at the same time.’ (Participant 2, individual interview)

## Perceived disadvantages of referral to a primary healthcare clinic

Six categories contributed to the overall category of perceived disadvantages of referral to a PHCC. They are concerns regarding the availability of medicines at PHCCs, the availability of medical practitioners and specialists at primary healthcare level, losing established relationships with hospital healthcare workers in a familiar setting, waiting times at PHCCs, mistreatment by nursing staff at PHCCs, and concerns regarding stigmatisation and lack of confidentiality at local clinics.

### Concerns regarding the availability of medicines at primary healthcare clinics

Participants were in general very concerned about medicines frequently being unavailable at PHCCs. It happened that some participants experienced adverse outcomes as a result of medicines not being available. One participant lamented:

‘One month I’ll receive the correct medication at the clinic, the next month all my medication is out of stock so I am not given any treatment, and the following month they put me on different medication because my medication is still not available.’ (Participant 56, mini-essay)

Another participant complained:

‘I was once without my treatment for 5 months because they had no stock at the clinic. As a result I relapsed and had to be re-admitted [*to hospital*].’ (Participant 5, individual interview)

Another participant remarked:

‘Sometimes they told me to come back another day and then, when you arrive, you are told that they are still waiting for stock. You then have no other choice than turning to alcohol to ease your depression, tension and stress.’ (Participant 11, individual interview)

As a last example, a participant, who reported being lucky to be back at WKH, said:

‘They told me that I will have to buy my treatment from a private pharmacy. I had to pay out of my own pocket and my medication is extremely expensive. Patients like me that only receive a [*disability*] grant can’t afford to buy treatment privately. Luckily I am now back at WKH and receiving my monthly medication.’ (Participant 1, individual interview)

### Concerns regarding medical practitioners and specialists at primary healthcare clinics

Participants reported that at some PHCCs there are no medical practitioners. Consequently patients are treated by nursing staff. Many participants held the view that medical practitioners are better equipped to manage mental disorders than nursing staff. According to one participant:

‘I think that coming to WKH is a good thing unlike going to the clinic where you will only be seen by a nurse … the nurses won’t be able to help you like the doctors in the hospital [*WKH*].’ (Participant 51, mini-essay)

Other participants were concerned that medical practitioners at some PHCCs can only be consulted on an appointment basis. This was a concern, because treatment might need urgent adjustment. For example:

‘At my clinic the doctor is not available every day, you can only see him on an appointment basis … when you feel unwell you can’t just see the doctor, you have to wait for your appointment. Sometimes he also just didn’t pitch for appointments. Then they [*nursing staff*] just repeated my same treatment for another month. I prefer to follow up here [*WKH*], it’s much better for me than going to my clinic where I never know when the doctor will be available.’ (Participant 13, individual interview)

Many participants were very concerned about the fact that they would not be treated by a specialist at the PHCC. In their view, mental healthcare should be rendered by healthcare workers with the necessary training, experience and skills, and consequently an institution like WKH is preferred:

‘ … if there are problems with my treatment I can immediately discuss it with my psychiatrist at WKH … I think the training of the nurses and doctors at local clinics are not adequate to manage people with psychiatric problems. I don’t think that they have the necessary experience.’ (Participant 55, mini-essay)

The comments of some participants indicated a real unwillingness to be referred to a PHCC, because of the reported absence of specialists in mental health:

‘WKH is a specific institution that renders a service for a particular illness which need[*s*] critical attention at all times … the service rendered here at WKH cannot be compared to that of my local clinic. As a result my mental health will be at risk … if I had the power to stop referral I would exercise it.’ (Participant 7, mini-essay)

### Concerns regarding loss of established relationships with hospital healthcare workers in a familiar setting

Participants valued the trusting relationships that they had formed with healthcare workers at WKH. They were of the opinion that it would be difficult and distressing to start over in a different, unfamiliar setting. For example:

‘I have built a trusting relationship with my doctors and the staff [*at WKH*] … trusting people with your problems is a major issue. Therefore, being referred could adversely influence my recovery. I would have to build new relationships in order to gain trust – this will slow down my recovery. I feel more comfortable and relaxed here [*at WKH*] …’ (Participant 8, individual interview)

Sometimes a clear fear of the unknown (the PHCC) surfaced: ‘I am used to the setting [*at WKH*] … it will be too stressful and difficult to change to a new place’ (Participant 17, mini-essay).

### Concerns regarding waiting times at primary healthcare clinics

Waiting times at PHCCs seem to be an issue, because ‘you have to wait long to be seen at the clinic …’ (Participant 32, mini-essay).

Sometimes: ‘… you wait the whole day until the clinic closes at 4 o’clock and you are told to come back the next day’ (Participant 13, individual interview).

Participants were of the opinion that the reason for these delays is ‘… there are just too many people at the clinic’ (Participant 11, individual interview).

### Concerns regarding poor treatment by nursing staff at primary healthcare clinics

It was disconcerting to learn that, from the participants’ point of view:

‘The way the staff at the clinics treat you makes you really feel as if you are worth nothing … they have a very hostile attitude towards patients … there is a big problem, they should really do something about that … they treat you really badly.’ (Participant 14, individual interview)

This in turn led participants to report: ‘The staff at the clinic treats me in a negative manner and they are not helpful … I prefer to rather travel to WKH for treatment’ (Participant 26, mini-essay).

### Concerns regarding stigmatisation and lack of confidentiality at local clinics

For some participants stigmatisation is a sensitive issue. Consequently:

‘I know people in my community that attend my local clinic, if they see me at the clinic they will ask me what I am doing there. What will they think of me if they hear that I am receiving treatment for mental illness? I will have to carry the stigma. I can’t bear that.’ (Participant 49, mini-essay)

Also:

‘When they think about mental illness they start thinking that you are crazy and they start treating you as if you are crazy … They start talking behind your back about how crazy you are.’ (Participant 4, individual interview)

This sensitivity about stigmatisation is sometimes compounded when there is a perceived lack of confidentiality. This happens when ‘[*the nurses*] will start talking about the fact that you are taking medication for a mental problem. They gossip about it’ (Participant 3, individual interview).

In contrast to the perceived stigmatisation at clinics, at WKH, there ‘… is no stigmatisation [*of patients*] by staff’. Also, ‘at WKH the patients all suffer from mental illness. They don’t judge each other’ (Participant 10, mini-essay).

## Willingness to be referred if concerns are addressed

Many participants were willing to be referred to PHCCs if their concerns were addressed:

‘We would love to be able to receive service [*mental healthcare*] at our clinic, but then there has to be a psychiatrist, a wide range of psychiatric medications and the guarantee that our medication will be in stock each month.’ (Participant 16, mini-essay)

Another participant mentioned:

‘referral can be very advantageous if some prerequisites are met … the clinic must make sure that I get the correct treatment every month … I will also need to be able to see a doctor regularly … they have to be better organised to reduce waiting times.’ (Participant 31, mini-essay)

A participant who was previously referred to a PHCC expressed willingness to retry referral if ‘the clinic can promise me that they will have my treatment in stock every month’ (Participant 23, mini-essay).

## Discussion

Participants were mostly of the opinion that the perceived disadvantages of referral to a PHCC outweighed the perceived advantages. Most participants were opposed to being referred because of the following reasons: unavailability of medicines, lack of doctor-based care, lack of specialised care, loss of established relationships with familiar healthcare workers at WKH, mistreatment by nursing staff at PHCCs, longer waiting times and higher levels of stigmatisation. Concerns regarding mistreatment and stigmatisation are serious: this would be in direct violation of mental healthcare users’ rights as enshrined in the *Mental Health Care Act*, No. 17 of 2002, specifically Sections 8 and 10. Section 8 is about respect, human dignity and privacy, while Section 10 addresses unfair discrimination.^9^ The importance of these two sections and how they were disregarded regarding institutionalised mental healthcare users was recently highlighted by the Office of the Health Ombud, in ‘The Report into the Circumstances Surrounding the Deaths of Mentally Ill Patients: Gauteng Province’.^19^

This negative view about referral to a PHCC is of concern, because, in South Africa, mental healthcare was planned to be integrated into the whole package of healthcare services delivered at a primary healthcare level. This model is promulgated to be a people-orientated healthcare system.^3,9^ Ironically, participants in this research were of the opinion that their opinions, or specific healthcare needs, were not being taken into account.

Instead of welcoming the possible advantages of referral to a PHCC, which were recognised by participants, referral to a PHCC was generally viewed as something negative that may even adversely affect a participant’s mental health, for example, by relapsing or turning to substances for relief.

The South African Primary Healthcare Level Standard Treatment Guidelines and Essential Medicines List^3,20^ makes provision for a limited range of psychiatric drugs for primary healthcare. It may be so that some patients are referred to a PHCC from a secondary or tertiary healthcare facility without due consideration for the drugs available at a PHCC, but whether that is so is not answered by this study. Yet it emerged that participants complained about drugs that should have been available but that had not been available.

In the primary healthcare model, nursing staff is largely responsible for the management of patients’ care.^2,3^ Participants tended to prefer treatment by a specialist at a service delivery point that focuses exclusively on the management of persons suffering from mental disorders. That was so not only for the skill level of mental healthcare workers but also to avoid stigmatisation at a generalist PHCC. The latter is a somewhat surprising counter claim to what the WHO claims, namely that integration into primary healthcare reduces stigmatisation.^2^

Not only did participants have an issue with the skills of primary healthcare workers and stigmatisation at PHCC, they also alleged that PHCC nursing staff treated them badly. Being ill-treated is not only unpleasant but unsettles the therapeutic relationship, which in turn is important for compliance to treatment.^21^ If there is a perception of uncaring treatment of mental health patients by PHCC staff, it will compound a fear that may already be present: that is, as expressed by participants, the loss of follow-up treatment in a familiar hospital environment perceived as being caring.

Despite the perceived disadvantages, participants were not blind to the possible advantages. It is important to note that participants were not completely set against being referred to a PHCC. They expressed a clear willingness to be referred if their concerns regarding the care they receive at PHCC level were addressed.

There are a number of limitations to this study. Firstly, these findings pertain to, and are limited to, patients attending WKH and are therefore not generalisable to other settings. Secondly, the study was not about any specific PHCC. Consequently, the negative opinions expressed by participants cannot be generalised to any specific clinic or group of clinics. Thirdly, the views are those of the participants only – the study did not seek to clarify related views by healthcare workers. Even so the opinions of the study participants should not be discarded but remedied as would be appropriate. Lastly, all participants in this study were patients following up at WKH, and consequently they may have been disinclined to express negative opinions about their current healthcare providers.

## Conclusion

The findings of this study contribute to a better understanding of patients’ opinions about, and experience of, referral. The successful integration of mental healthcare into primary healthcare in South Africa will only be possible if more attention is given to the opinions and concerns of the users of these healthcare services. Thus, there is a need for more research in this area in order to elucidate more accurately the discrepancies between policy imperatives and the actual level of care received. Such research may build on this research by using a qualitative approach or may use research like the present one to develop quantitative tools for future quality assurance and research.
